# Supplementary material for: A Description and Safety Overview of Irreversible Electroporation for Prostate Tissue Ablation in Intermediate-Risk Prostate Cancer Patients: Preliminary Results from the PRESERVE Trial
Source: Cancers (Basel). 2024 Jun 8;16(12):2178. doi: 10.3390/cancers16122178 (PMC11201469; doi:10.3390/cancers16122178)
Supplement: Supplementary file 1 [file cancers-16-02178-s001.zip › Supplementary Figure S1.pdf]

Supplementary Figure S1. The NanoKnife System and NanoKnife System Probes

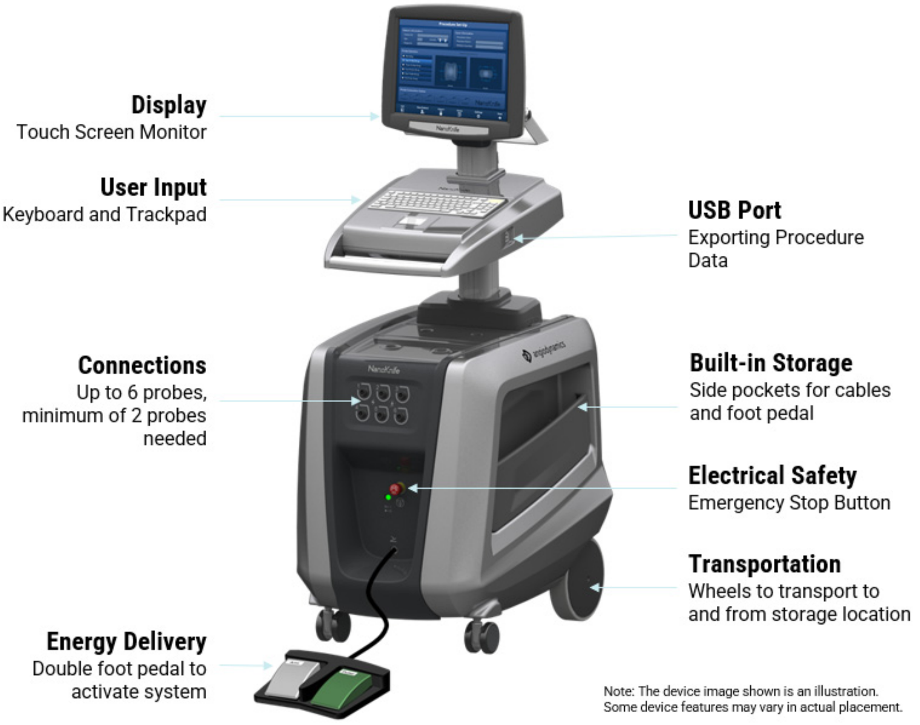

(a) The NanoKnife System Generator

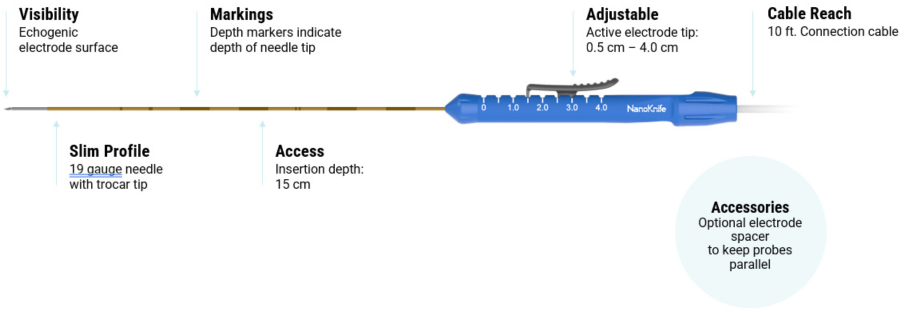

(b) The NanoKnife Single Electrode Probe
