# Supplementary material for: A Description and Safety Overview of Irreversible Electroporation for Prostate Tissue Ablation in Intermediate-Risk Prostate Cancer Patients: Preliminary Results from the PRESERVE Trial
Source: Cancers (Basel). 2024 Jun 8;16(12):2178. doi: 10.3390/cancers16122178 (PMC11201469; doi:10.3390/cancers16122178)
Supplement: Supplementary file 1 [file cancers-16-02178-s001.zip › Supplementary File S1.pdf]

Supplementary File S1. Study Eligibility Criteria for the PRESERVE Trial

A subject was required to fulfill all of the following criteria to be included in the study:

1. Is greater than 50 years of age
2. Has at least a 10-year life expectancy
3. Has histologically confirmed organ-confined prostate cancer, clinical stage ≤ T2c
4. Has a prostate-specific antigen (PSA) ≤ 15 ng/mL or PSA density < 0.15 ng/mL^2 if PSA is > 15 ng/mL
5. Has Gleason score 3+4 or 4+3
6. Has no evidence of extraprostatic extension by multiparametric MRI (mpMRI)
7. Has no evidence of seminal vesicle invasion by mpMRI, and if suspected, confirmed by biopsy
8. Physician is able to visualize prostate gland adequately on transrectal ultrasound imaging during qualifying biopsy
9. Has a transperineal or transrectal targeted prostate biopsy of lesion, plus 10-14 core systematic biopsy to include adequate sampling of the peripheral zone correlating with an intermediate risk lesion[1] in the area of the MR-visible lesion
10. A visible lesion on mpMRI that is accessible to irreversible electroporation (IRE) treatment (Note: prostate cancer detected via systematic standard biopsy outside of the adjacent sextant location of the MRI visible lesion will meet entry criterion provided the positive core is Gleason 6; has fewer than 3 prostate biopsy fragments/cores positive and ≤50% cancer in each of those fragments/cores on standard biopsy)
11. Is willing and able to sign a written informed consent and in the judgment of the physician, the study is in the best interest of the subject
12. Understands and accepts the obligation and is logistically able to present for all scheduled follow-up visits

A subject was excluded from the study if they met any of the following criteria:

1. Has known hypersensitivity to pancuronium bromide, atricurium or cisatricurium
2. Is unfit for anesthesia or has a contraindication for agents listed for paralysis
3. Has an active urinary tract infection (UTI)
4. Has a history of bladder neck contracture
5. Is interested in future fertility
6. Has a history (within 3 years) of inflammatory bowel disease
7. Has a concurrent major debilitating illness
8. Had active treatment for a malignancy within 3 years, including malignant melanoma, except for prostate cancer or other types of skin cancer
9. Has any active implanted electronic device (e.g., pacemaker)
10. Is unable or unwilling to catheterize
11. Has had any prior or current prostate cancer therapy, including:
    a) Biologic therapy for prostate cancer
    b) Chemotherapy for prostate cancer
    c) Hormonal therapy for prostate cancer within three months of procedure
    d) Radiotherapy for prostate cancer

---

[1] An intermediate risk lesion is defined as Gleason score 3+4 or 4+3, PSA < 15 ng/mL or PSA density < 0.15 ng/mL^2 if PSA is > 15 ng/mL, and ≤ clinical stage T2c.

e) Surgery for prostate cancer

12. Has had prior transurethral prostatectomy (TURP), stricture surgery, urethral stent or prostatic implants
13. Has had prior major rectal surgery (except hemorrhoids)
14. Is unfit for pelvic MRI scanning (e.g., severe claustrophobia, permanent cardiac pacemaker, metallic implants that are likely to contribute significant image artifacts, allergy or contraindication to gadolinium (to enhance MRI))
15. Is actively bleeding, has a bleeding disorder, or is unable to interrupt blood thinning medications as clinically indicated per pre-operative best practices
16. Is a member of a vulnerable population, such as cognitively impaired or incarcerated, that could expose them to undue influence, coercion, or inability to obtain informed consent
17. In the opinion of the treating physician, has a contraindication listed in the current NanoKnife System User Manual (section 2.3)
