# Supplementary material for: A Description and Safety Overview of Irreversible Electroporation for Prostate Tissue Ablation in Intermediate-Risk Prostate Cancer Patients: Preliminary Results from the PRESERVE Trial
Source: Cancers (Basel). 2024 Jun 8;16(12):2178. doi: 10.3390/cancers16122178 (PMC11201469; doi:10.3390/cancers16122178)
Supplement: Supplementary file 1 [file cancers-16-02178-s001.zip › Supplementary File S2.pdf]

Supplementary File S2. Schedule of Subject Evaluations

Study Assessment	**Visit 1**	**Visit 2**	**Visit 3**	Post-Procedure[10]	**Visit 4**	**Visit 5**	**Visit 6**	**Visit 7**	**Visit 8**
	Screening	Baseline[3]	NanoKnife Treatment		1 mo<br>30 ± 7 days	3 mos<br>90 ± 14 days	6 mos<br>180 ± 14 days	9 mos<br>270 ± 14 days	12 mos<br>365 ± 28 days
Informed Consent[1]	X								
Enrollment	X								
Medical History	X								
Standard Physical Exam		X			X	X	X	X	X
Abbreviated Physical Exam			X						
Vitals Assessment[4]		X	X		X	X	X	X	X
CBC Panel		X							
Prothrombin Test (PT)		X							
Partial Thromboplastin Time (PTT)		X							
Urinalysis		X	X[9]	X[5]	X[5]	X[5]	X[5]	X[5]	X[5]
Transrectal US	X		X						X
MRI protocols	X[2]			X[6]		X			X
Biopsy[7,8]	X[2]								X
PSA	X[2]				X	X	X	X	X
UCLA-EPIC Urinary Questionnaire		X			X	X	X	X	X
IPSS & IPSS-QoL Questionnaire		X			X	X	X	X	X
IIEF-15 Potency Questionnaire		X			X	X	X	X	X

EQ-5D Questionnaire		X			X	X	X	X	X
NanoKnife System Procedure			X						
Concomitant Medications		X	X	X	X	X	X	X	X
Adverse Event Assessment			X	X	X	X	X	X	X

1. Must be signed and dated prior to completing any study procedures.
2. Must be completed within 180 days prior to enrollment in the study. PSA for enrollment must be completed prior to biopsy, or must be taken more than 30 days after biopsy.
3. Complete within 30 days prior to NanoKnife System treatment.
4. Vitals assessments include height and weight, temperature, BP, respiration rate and pulse. Note, height only needs to be collected at Baseline.
5. Complete only if there is a question of an active UTI.
6. Complete only if clinically indicated (i.e., the subject is experiencing unexpected adverse events following treatment).
7. Transperineal or Transrectal Prostate Biopsy (template mapping and/or limited targeted).
8. Complete at additional time points if subject has a lesion ≥ PI-RADS 3 on the 3-month MRI, experiences biochemical failure as defined by the Phoenix criteria as nadir + 2 ng/mL, or upon clinical suspicion.
9. Complete pre-treatment, and at discharge only if there is a question of an active UTI.
10. Complete within 3 to 10 days of NanoKnife System treatment. This visit can be conducted over the phone, as long as urinalysis and MRI are not clinically indicated.
