# Supplementary material for: A Description and Safety Overview of Irreversible Electroporation for Prostate Tissue Ablation in Intermediate-Risk Prostate Cancer Patients: Preliminary Results from the PRESERVE Trial
Source: Cancers (Basel). 2024 Jun 8;16(12):2178. doi: 10.3390/cancers16122178 (PMC11201469; doi:10.3390/cancers16122178)
Supplement: Supplementary file 1 [file cancers-16-02178-s001.zip › Supplementary Table S1.pdf]

Supplementary Table S1. Demographics and Disease Characteristics

Characteristic		Study Population N=121
Race [n (%)]	Asian	7 (5.8)
	Black or African American	10 (8.3)
	White	96 (79.3)
	Other	7 (5.8)
	Unknown	1 (0.8)
Ethnicity [n (%)]	Hispanic or Latino	4 (3.3)
	Not Hispanic or Latino	110 (90.9)
	Not Reported	3 (2.5)
	Unknown	4 (3.3)
Age at Informed Consent (years)	N	121
	Mean (std)	66.6 (7.16)
	Median	67.0
	Q1, Q3	61.0, 72.0
	Min, Max	49.0, 82.0
Age Group [n (%)]	<65 years	47 (38.8)
	>=65 years - <75 years	56 (46.3)
	>=75 years	18 (14.9)
AJCC Primary Tumor Stage at Screening [n (%)]	n	119
	T1b	1 (0.8)
	T1c	103 (86.6)
	T2a	11 (9.2)
	T2b	4 (3.4)
Gleason Score at Screening [n (%)]	n	121
	3+4	97 (80.2)
	4+3	24 (19.8)
